# A Portable and Power-Free Microfluidic Device for Rapid and Sensitive Lead (Pb^2+^) Detection

**DOI:** 10.3390/s120709467

**Published:** 2012-07-10

**Authors:** Chunhui Fan, Shijiang He, Gang Liu, Lianhui Wang, Shiping Song

**Affiliations:** 1 Laboratory of Advanced Materials, Fudan University, 2205 Songhu Road, Shanghai 200438, China; E-Mail: 09213010009@fudan.edu.cn; 2 Laboratory of Physical Biology, Shanghai Institute of Applied Physics, Chinese Academy of Sciences, Shanghai 201800, China; E-Mail: heshijiang@sinap.ac.cn; 3 Shanghai Institute of Measurement and Testing Technology, Shanghai 201203, China; E-Mail: liug@simt.com.cn

**Keywords:** gold nanoparticles, Pb^2+^, 11-mercaptoundecanoic acid, microfluidic chip

## Abstract

A portable and power-free microfluidic device was designed for rapid and sensitive detection of lead (Pb^2+^). 11-mercaptoundecanoic acid (MUA)-functionalized gold nanoparticles (MUA-AuNPs) aggregated in the presence of Pb^2+^ for the chelation mechanism. When we performed this analysis on a polydimethylsiloxane (PDMS) microfluidic chip, the aggregations deposited onto the surface of chip and formed dark lines along the laminar flows in the zigzag microchannels. This visual result can be observed by the naked eye through a microscope or just a drop of water as a magnifier. Ten μM Pb^2+^ was successfully detected.

## 1. Introduction

Heavy metal ions (HMIs), such as lead (Pb^2+^), mercury (Hg^2+^) represent significant hazards to the environment and human health [1–4], since they can contaminate the soil and water and enter the water supply and food chain. Their residues and toxicity cause serious and long-term effects. Thus development of rapid and sensitive methods for the detection of HMIs has become a very urgent need and has drawn a lot of research interest in recent years.

So far, many techniques have been developed to detect HMIs such as atomic absorption/emission spectroscopy (AAS), inductively coupled plasma-mass spectrometry (ICP-MS) and mass spectroscopy (MS) [5–7]. However, most of the existing methods rely heavily on expensive and complicated techniques, thus fast and simple sensors are in great demand.

Gold nanoparticles (AuNPs) have been extensively explored as sensing probes because of their unique optical and electrochemical properties [8]. Since Mirkin *et al.* [9,10] first reported that AuNPs' aggregation is accompanied by a color change, colorimetrical assay methods utilizing AuNPs have been widely developed. Xia *et al.* achieved a universal colorimetric assay employing gold nanoparticles and water-soluble conjugated polyelectrolytes, including nucleic acid, small molecules, proteins, and inorganic ions [11], while Wang *et al.*, developed a platform for the detection of magnesium and pyrophosphate ions [12]. Besides, colorimetric analysis have also made progress in the detection of viruses and bacteria [13,14]. Recently, the application of modified AuNPs in HMI detection has attracted considerable research interest. For example, Lu's group developed a sensor based on the Pb^2+^-dependent DNAzyme and AuNPs [15]. In the presence of Hg^2+^, oligo-T can form a thymine-Hg^2+^-thymine (T-Hg^2+^-T) complex, thus many novel Hg^2+^ detection approaches have been developed based on oligo-T modified AuNPs [16,17].

More recently, 11-mercaptoundecanoic acid (MUA) functionalized gold nanoparticles have been demonstrated as good probes for the detection of HMIs in aqueous solution by Hupp *et al.* [18]. Compared to other AuNP probes, MUA modified AuNPs are simple and cheap to produce, which suggests their huge potential for practical applications. In our previous work [19], a portable device based on power-free PDMS microfluidic technology has been developed. The sensor can detect mercury ions with great sensitivity with the naked eyes, which shows great practicality in analysis of actual samples.

**Figure 1.**
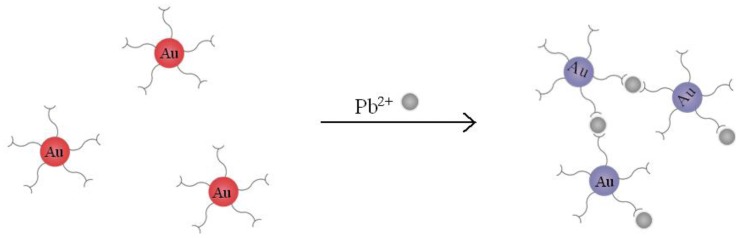
Schematic illustration of the chelation mechanism of Pb^2+^ ions and MUA-AuNPs.

In this work, we detected Pb^2+^ with AuNPs which are modified by MUA (MUA-AuNPs). As probes, The chelation between MUA and Pb^2+^ will cause the aggregation of the MUA-AuNPs. We can obviously see the solution color changed from red to purple caused by the effect of plasmonic coupling (Figure 1). While on our power-free PDMS microfluidic device, the aggregations thus formed deposited onto the surface of PDMS, resulting in a dark line which can be observed under a microscope.

## 2. Materials and Methods

### 2.1. Reagents and Materials

**Figure 2.**
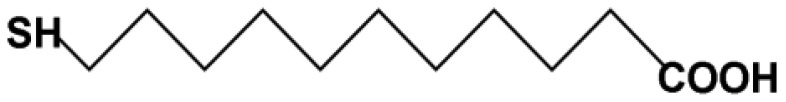
11-Mercaptoundecanoic acid (MUA) molecule (C_11_H_22_O_2_S).

PDMS (Sylgard 184) was purchased from Dow Corning (Midland, MI, USA). Chloroauric acid trihydrate (HAuCl_4_·3H_2_O), HMIs and 11-mercaptoundecanoic acid (MUA, Figure 2) were obtained from Sigma (St. Louis, MO, USA). Deionized water (18.2 MΩ) produced by the Milli-Q system was used throughout the experiments.

### 2.2. Preparation of Au Nanoparticles

Fifteen (15) nm Au nanoparticles (AuNPs) were prepared by citrate reduction of HAuCl_4_ which is similar to Grabar's method [20]. 1% trisodium citrate solution (4 mL) was added to a boiling solution of HAuCl_4_ (99 mL deionized water and 1 mL 1% HAuCl_4_). The mixture was kept boiling and stirring for about 30 min until the color of the aqueous change from yellow to red. After that, the solution was cooled to room temperature while being stirred continuously and then the prepared AuNPs was stored at 4 °C.

### 2.3. Modification of AuNPs

The modification of AuNPs with 11-mercaptoundecanoc acid (MUA-AuNPs) was carried out mainly as reported [18] with some changes as follows: aqueous solution (500 μL) consisting of MUA (2.4 mM) and an equivalent amount of sodium hydroxide (2.4 mM) were added to 15 nm Au nanoparticle suspension (500 μL). The mixed solution was stirred with the speed of 450 rpm in 80 °C for 1 h on the Thermo mixer (Eppendorf, Hamburg, Germany). After cooling down to room temperature, the mixture was centrifuged twice (10,000 rpm, 10 mins, 4 °C) and the supernatant was replaced with deionized water.

### 2.4. Fabrication of Microfluidic Chips

Microfluidic chips with Y shape and zigzag microchannels were firstly fabricated according to standard photolithographic methods [21]. Then a negative master was prepared on the silicon wafer by SU-8 photoresist, and a plasma etcher was used to realize the passivation of the master. The prepared master was placed in a glass bottom dish. After that, PDMS prepolymer (10:1 v/v mixture) was degassed and cast onto the master. After heating for 2 h at 80 °C, the PDMS was removed from the substrate and the holes punched with corresponding metal pipes. We used a flat PDMS slab (3 mm thick) as the substrate that bonding with the prepared PDMS layer to form the channels for chemical reaction. The dimensions of the finished microchannels in the chip are 100 μm (width) × 30 μm (height), and the whole chip are 3.5 cm (width) × 5 cm (length).

### 2.5. Preparation and Inletting of the Regent

After degassing at 10 kPa for 1 h, the microfluidic chip was immediately sealed with adhesive tape except for the inlet reservoirs to be ready for Pb^2+^ detection. The MUA modified AuNPs and different test samples were added into two inlet reservoirs using micropipette.

## 3. Results and Discussions

### 3.1. Colorimetric Analysis in Solution

**Figure 3.**
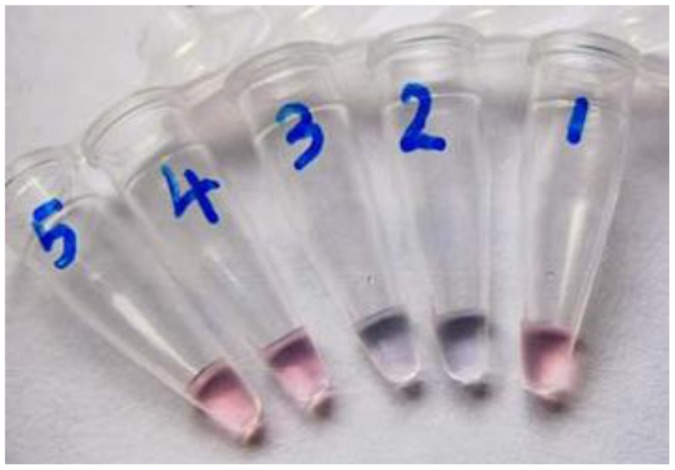
Photographic image of MUA-AuNPs, with their colour visibly changed in the presence of Pb^2+^. (1) Deionized water; (2) 0.05 mM Pb^2+^; (3) 0.025 mM Pb^2+^; (4) 0.01 mM Pb^2+^; (5) 0.005 mM Pb^2+^.

Our AuNP-based HMI sensor was based on the colour change caused by the surface plasmon resonance effect on AuNP aggregation. It is known that normal AuNPs with diameter between 10 nm to 50 nm appear red in colour in water [22,23], as we can see from the tube 1 in Figure 3, when Pb^2+^ was added, chelate interaction with the carboxylate groups of MUA-AuNPs occurred, which shortens the distance between the gold nanoparticles and formed aggregates. Owing to the inter-particle coupled plasmon excitons in the aggregated states, a red-to-purple color change can be observed in the solutions. As shown in Figure 3, the colorimetric response to Pb^2+^ resulted in an obvious color change in the number two and three tubes. We challenged the strategy with other metal ions, including Mn^2+^ and Zn^2+^, and the experimental results demonstrated that Pb^2+^ had a much stronger signal (data not shown). Yet to be improved, an obvious megascopic response can only be obtained with concentrations higher than 0.025 mM in solution.

### 3.2. Au-MUA's Deposition in the Microfluidic Channels Induced by Pb^2+^

**Figure 4.**
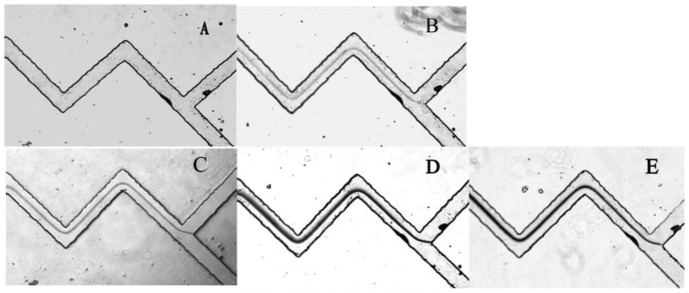
Microscope images of the analysis results for different concentration of Pb^2+^: (A) Deionized water; (B) 10 μM Pb^2+^; (C) 25 μM Pb^2+^; (D) 50 μM Pb^2+^;(E) 100 μM Pb^2+^.

PDMS has been widely used in microfluidic systems for their optical transparency, low toxicity and ease of fabrication properties [24,25]. More importantly, with its high gas solubility, PDMS was demonstrated to be a great potential material for power-free microchips [26]. The equilibrium concentration of gas dissolved in PDMS is directly proportional to the local gas pressure around the PDMS. Accordingly, when a vacuum degassed PDMS device returns to the atmosphere, it will absorb air to establish new state of equilibrium which will automatically cause a negative pressure in the microfluidics channels. This is the basic principle of a power-free PDMS device. Our PDMS chip was fabricated with two Y-shaped zigzag micro-channels, and the two inlets were at the equal position of the upper corners of Y shape [19]. Zigzag shaped channels were designed to favour solution mixing. The PDMS microfluidic chip was firstly degassed in an airtight vacuum desiccator for 1 h, then 3 μL of MUA-AuNPs solution and 3 μL of different concentrations of Pb^2+^ (or deionized water, unknown samples) were added into each inlet reservoir. Drawn by the negative pressure in the channels, the droplets from two inlets simultaneously flowed to the junction and mixed together in the channels. Since the passages have twists and turns these enhanced the degree of mixing, and MUA-AuNPs and Pb^2+^ reacted sufficiently. Carboxylate groups on the surface of MUA-AuNPs chelated Pb^2+^, and made the nanoparticles aggregate. Gradually, a dark line can be observed using microscope in the presence of Pb^2+^ whereas no line can be seen without Pb^2+^. As we can see from Figure 4, the Pb^2+^ solution with a concentration as low as 10 μM could form a very clear line, while the blank samples generated no visual signal (Figure 4A). The time for solution flowing from inlet to outlet reservoir was less than 15 min, which is a remarkable advantage for real use. It was demonstrated to be a simple, fast and feasible method which showed great superiority of the power-free microfluidic device.

**Figure 5.**
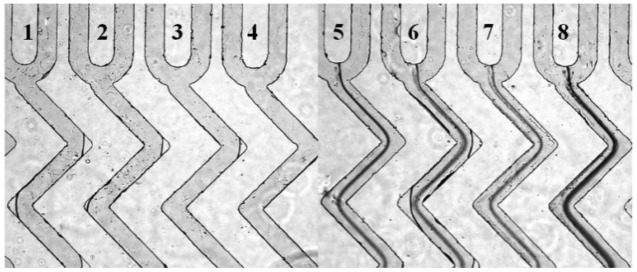
Microscope images of the lines in multi-microchannels microfluidic chip in the presence of different ion solutions. (1) 100 μM Mg^2+^; (2) 100 μM Ca^2+^; (3) 100 μM NaCl; (4) Deionized water; (5) 10 μM Pb^2+^; (6) 25 μM Pb^2+^; (7) 50 μM Pb^2+^; (8) 100 μM Pb^2+^.

With a high throughout microfluidic chip that include eight microchannels, different concentrations of HMIs can be detected simultaneously. The process of sample injection was the same with the operation of the single microchannel microfluidic chip. The same analysis effect was achieved even on a multi-microchannel microfluidic chip. As shown in Figure 5, the channels numbered from 5 to 8 that contained Pb^2+^ displayed obvious dark lines. In contrast, the control ions, such as Na^+^, Ca^2+^ and Mg^2+^ did not show any aggregation (lines 1–4 in Figure 5).

**Figure 6.**
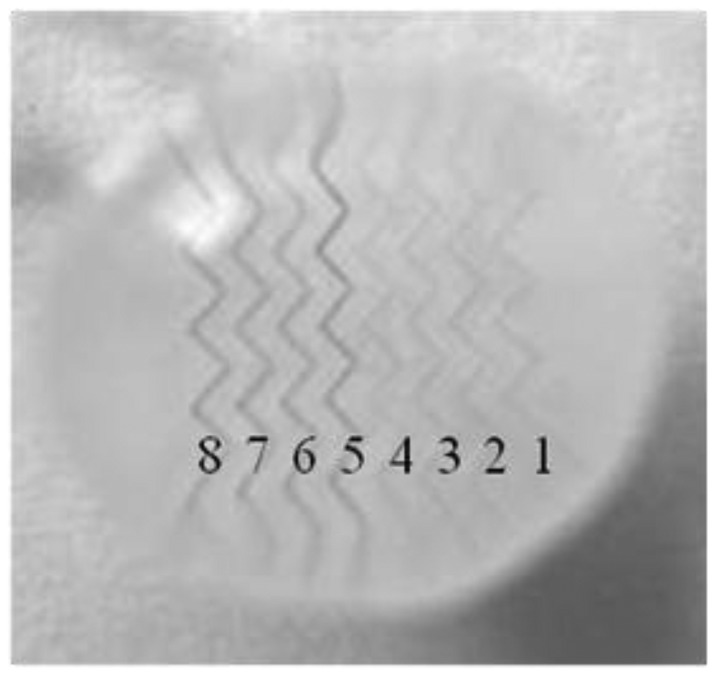
Image taken by a camera through a drop of water on a microfluidic chip. Channel 1–8 showed the analysis result from samples of different concentrations of various ions corresponding to the results shown in Figure 4: (1) Deionized water; (2) 0.1 mM NaCl; (3) 0.1 mM Ca^2+^; (4) 0.1 mM Mg^2+^; (5) 0.1 mM Pb^2+^; (6) 0.05 mM Pb^2+^; (7) 25 μM Pb^2+^; (8) 10 μM Pb^2+^.

Interestingly, just with the help of a drop of water as a magnifier, the lines can be seen with the naked eye. As shown in Figure 6, the microchannels can be identified clearly under a normal camera. Therefore we can read out the results easily due to the visible aggregation lines, which is quite important for the field testing.

## 4. Conclusions

In summary, we have developed a simple and portable Pb^2+^ sensor on a microfluidic chip with a detection limit of 10 μM Pb^2+^ which is competitive with traditional physico-chemical quantitative methods. Among the numerous methods that focus on metal ions detection, such as fluorescence and colorimetric methods [27–29], electrical detection based on reduced grapheme [30], surface enhanced Ramans cattering (SERS) sensors [31,32] and so on, our device has obvious advantages. Firstly, it is considearbly more convenient because it is power-free and reusable, and we can read the analysis results with our naked eyes through just a water drop as a magnifier. Secondly, it is an effective and fast detection method, that the whole detection process can be completed in less than a quarter hour. Thirdly, it is a low-cost analysis because no expensive and complicated reagents or equipment are needed. Thus, we believe our strategy offers a Pb^2+^ sensor with great potential popularity, and with proper probes and condition optimization, many other targets can be detected.
